# Identifying tumor antigens and immune subtypes of gastrointestinal MALT lymphoma for immunotherapy development

**DOI:** 10.3389/fonc.2022.1060496

**Published:** 2022-12-08

**Authors:** Xinlu Jiang, Huanhuan Zhang, Jinju Ni, Xu Zhang, Kaiyang Ding

**Affiliations:** ^1^ Department of Hematology, Anhui Provincial Hospital Affiliated to Anhui Medical University, Hefei, Anhui, China; ^2^ Department of Respiratory, Wannan Medical College, Wuhu, Anhui, China; ^3^ Department of Urology, The First Affiliated Hospital of Nanjing Medical University, Nanjing, China

**Keywords:** gastrointestinal MALT lymphoma, prognostic factors, KLHL14, mRNA vaccines, drugs target

## Abstract

MALT lymphoma is an extranodal B-cell lymphoma of the marginal zone of mucosa-associated lymphoid tissue (MALT), caused by malignant transformation of B-cells in the marginal zone. In this work, we aim to explore the potential relationship between MALT lymphoma and DLBCL. Vaccines derived from messenger ribonucleic acid (mRNA) may provide satisfactory results. Despite being a promising treatment option, immunotherapy isn’t widely used in treating renal cell carcinoma, as only a few patients respond to the treatment. The Cancer Genome Atlas (TCGA) analysis revealed gene expression profiles and clinical information. Antigen-presenting cells infiltrated the immune system using TIMER tool (http://timer.cistrome.org/). GDSC (Genomics of Drug Sensitivity in Cancer) data were used to estimate drug sensitivity. Immune-related genes were associated with a better prognosis in MALT lymphoma patients and higher levels of antigen-presenting cells. There is a significant relationship between these immune subtypes and immunological checkpoints, immunogenic cell death regulators, and prognostic variables for MALT lymphoma patients. In this study, we provide a theoretical foundation for the development of mRNA vaccines and suggest that KLHL14 could potentially be used as antigens to develop mRNA vaccines for MALT lymphoma.

## Introduction

MALT lymphoma is an extranodal B-cell lymphoma of the marginal zone of mucosa-associated lymphoid tissue (MALT), caused by malignant transformation of B-cells in the marginal zone (1). An acquired MALT in the stomach, usually caused by chronic Helicobacter pylori infection, is gastric MALT lymphoma (GML) (2). Most gastric lymphomas are MALT lymphomas, which make up 38%-48% of all primary gastric lymphomas (3). The majority of gastric MALT lymphomas are located locally and are usually of low grade (4). Although rare, MALT lymphoma has the risk of evolving into high-grade diffuse large B-cell lymphoma. It is believed that Helicobacter pylori played a significant role in the pathogenesis of gastric MALT lymphoma (5). A close relationship has been found between Helicobacter pylori infection and gastric lymphoma prevalence in epidemiological studies (6). It is important to eliminate H. pylori as soon as possible in the early stages of the condition, as successful eradication is usually sufficient to cure the condition (7). As far as we know, MALT lymphomas originate from mature post-germinal center B cells, which are closely related to plasma cells (8). There is, therefore, a significant proportion of lymphoma patients who express monoclonal immunoglobulins, both on the lymphoma cells and in the blood, which can be detected by serum electrophoresis or immunofixation (9).

Gastric MALT lymphoma is initially diagnosed using endoscopy and histological examination of biopsy specimens. The diagnosis of gastric MALT lymphoma is based on centrocyte-like B-cell infiltration in the lamina propria, and the histology reveals lymphoma cell aggregates infiltrating individual glands and forming prominent nodules (10). Clinical staging was determined using the modified Ann Arbor staging system, which classified major lesions according to their location in the upper, middle, or lower third of the stomach (11). Physical examinations, computed tomography scans of the chest and abdomen, and biopsies are all part of the staging process. Based on immunohistochemistry, MALT lymphoma cells typically express haplotype surface Igs, IgM (IgM1) is more prevalent than IgD1 and IgG1, and cytoplasmic Igs are present in 40% of cases (12). B-cell-associated antigens expressed by tumor cells include CD19, CD20, CD22, CD79a, and CD79b, along with CD5 negativity (CD52), CD432/CD431, CD32, CD232, and CD11c2/CD11c1 (13).

The non-Hodgkin lymphomas (NHLs) are a diverse group of malignancies that, in approximately 80% of cases, are caused by B cells (B-NHL) (14). B-NHL differs in presentation, clinical features, prognosis, and response to treatment. A majority of non-Hodgkin lymphoma (NHL) cases are diffuse large B-cell lymphomas (DLBCLs), which account for approximately one-third of the total number of NHL cases worldwide (15). During the last 40 years, CHOP (cyclophosphamide, doxorubicin, vincristine, and prednisone) has been widely used. Patients with DLBCL are treated with rituximab and CHOP in combination according to FDA guidelines approved in 2006 (16). In addition, immunotherapy has been widely used in the treatment of DLBCL patients in recent years. In the treatment of relapsed/refractory DLBCL, chimeric antigen receptor (CAR) T cells are emerging as a promising immunotherapy (17). Therefore, it is important to explore efficient immunotherapy for DLBCL patients.

## Methods

### Data sources

The mRNA expression profiles, as well as the clinical information of gastrointestinal MALT lymphoma, were obtained from the GEO database (https://www.ncbi.nlm.nih.gov/gds). In addition, the transcriptome expression data and clinical information of DLBCL patients were obtained from the TCGA database. In the GEO database, a total of four normal stomach tissues, five normal duodenal tissues, 10 gastric MALT lymphoma tissues, and 10 duodenal MALT lymphoma tissues were involved in the analysis. For the TCGA database, a total of 48 DLBCL tissues were included in the analysis.

### Screening of the differential expressed genes

The transcriptome data were divided into a patient group and a normal group. Differential expression analysis was performed using the “limma” package in R 4.1.1. Genes with P-value < 0.05 and |Log_2_FC|>=1 was defined as differentially expressed genes. The Venn Diagram is applied to obtain the genes involved in DLBCL and MALT lymphoma-related genes.

### GO and KEGG enrichment analysis

ClusterProfiler was used to analyze the functions of key genes. In addition, the Kyoto Encyclopedia of Genes and Genomes (KEGG) and Gene Ontology (GO) were used to explore the potential pathways involved in key genes. P-values lower than 0.05 were considered statistically significant.

### Protein-protein interaction network of TPP-related proteins

PPI network of interactive genes was performed in STRING (https://www.string-db.org/). Interaction with Composite Ratings of more than 0.4 was considered statistically significant. In addition, Cytoscape 3.8.2 was used to analyze and visualize PPI networks.

### Prognostic prediction model for DLBCL-related genes based prognostic index construction

The lasso regression analysis was performed to construct a prognostic prediction model. Subsequently, the screening of prognosis-related genes was further improved by applying SVM-based recursive feature elimination (SVM-RFE). All patients included in the prognostic model were assigned a risk score value. A grid search using 10-fold cross-validation yielded the best performance with the 40 top-ranked features. In addition, Kaplan-Meier survival analysis and receiver operating characteristic (ROC) curves were used to assess the accuracy of the prognostic model. AUC > 0.5 was considered to show predictive power.

### Gene set enrichment analysis

The gene sets were retrieved from the GSEA database (http://www.gsea-msigdb.org/gsea/downloads.jsp) and enriched GO terms and KEGG pathways were identified using GSEA analysis. A total of 50 best terms were selected for each subtype according to their significance.

### Classification of immune subtypes

Unsupervised NMF clustering of patients was performed using the NMF package based on the expression profile of immune-related genes. Further, “survival” the R software package was used for Cox regression analysis and analysis of the relationship between all candidate genes and overall survival (OS). Further, the NMF package was used for unsupervised NMF clustering, and the same candidate genes were used as the MSKCC external validation set. The k value at which the correlation coefficient began to decrease was selected as the optimal number of clusters. mRNA expression data of the above immune genes was used to verify the subtype assignment based on the T-SNE method.

### Drug sensitivity analysis

The “pRRophetic” R software package was used to predict the chemotherapy sensitivity of each tumor sample based on the Genomics in Drug Sensitivity in cancer Database ((GDSC), https://www.cancerrxgene.org/). Further, regression analysis was performed to determine the estimated IC50 value for each specific chemotherapy drug. The GDSC training set was used to perform ten-fold cross-validation to test the regression and prediction accuracy. Default values were selected for all parameters, including the “combat” parameter that removes batch effects and the average value of repeated gene expression.

### Immune cell infiltration analysis

The CIBERSORT algorithm was used to analyze RNA-seq data of PRAD patients in different subgroups to determine the relative proportions of 22 immune infiltrating cells. Subsequently, Spearman correlation analysis was performed to explore the relationship between gene expression and infiltration of the 22 immune cell types. A P-value <0.05 was considered statistically significant.

### Gene set variation analysis

GSVA is a non-parametric unsupervised method for evaluating the enrichment of transcriptome gene sets. In this analysis, gene-level changes are transformed into pathway-level changes by comprehensively scoring the gene set of interest, and the biological function of the sample is determined. In the present study, gene sets were retrieved from the molecular signatures database (v7.0 version). The GSVA algorithm was then used to comprehensively score each gene set to evaluate potential biological functional changes in different samples.

### Immune landscape analysis

Dimensionality reduction analysis was conducted using the ReduceDimension function in Monocle. The maximum number of components was set as four. The discriminative tree dimensionality reduction algorithm (DDRTree) was used for dimensionality reduction. PLOT_CELL_TRACTORK package in Monocle was used for visualization of the immune landscape.

### Gene set enrichment analysis

Gene sets were retrieved from the MSigDB database (http://www.gsea-msigdb.org/gsea/downloads.jsp). The R package was used to perform GSEA of the gene sets to identify enriched GO terms and KEGG pathways. The top 50 terms with the highest significance among the subtypes were selected.

### Statistical analysis

Survival curves were generated using the Kaplan-Meier method and were compared using the log-rank test. Cox proportional hazard model was used for multivariate analysis. All statistical analyses were performed using R (version 3.6) software. All statistical tests were two-sided, and a p-value <0.05 was considered statistically significant.

## Results

### Exploration of the key genes involved in the lymphoma cohort

**Figure 1:**
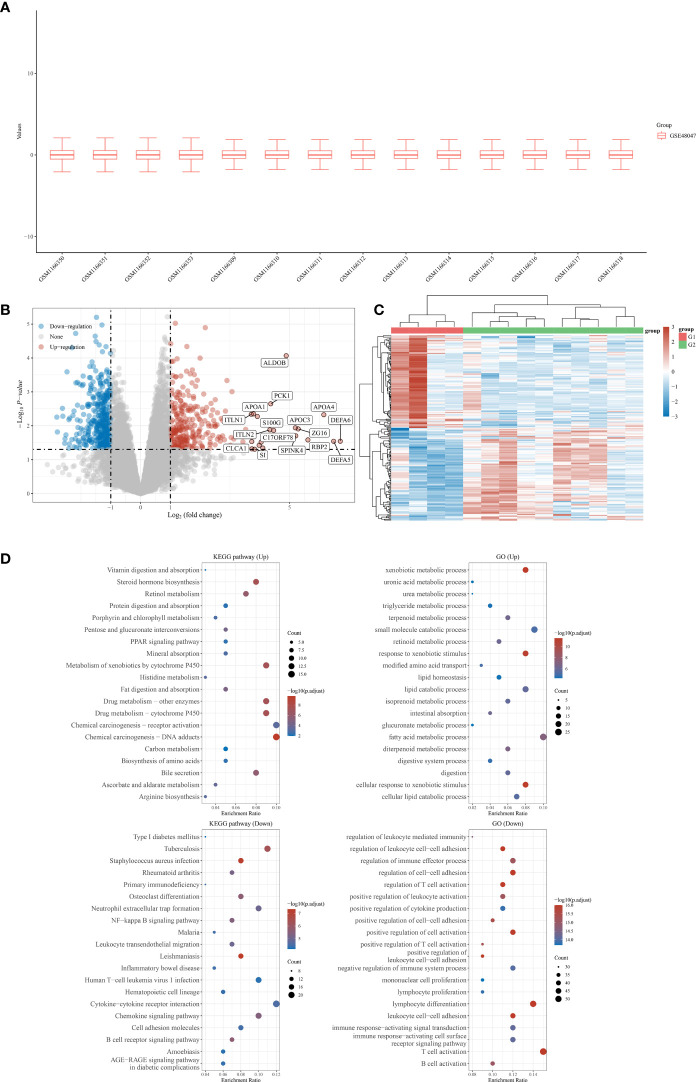
**(A)** The boxplot reveals the samples involved in gastric MALT lymphoma patients and control groups; **(B)** The differentially expressed analysis between gastric MALT lymphoma patients and normal people; **(C)** The heat map shows the top differentially expressed genes between gastric MALT lymphoma patients and normal people; **(D)** The GO and KEGG enrichment analysis based on the differentially expressed genes between gastric MALT lymphoma patients and normal people.

**Figure 2:**
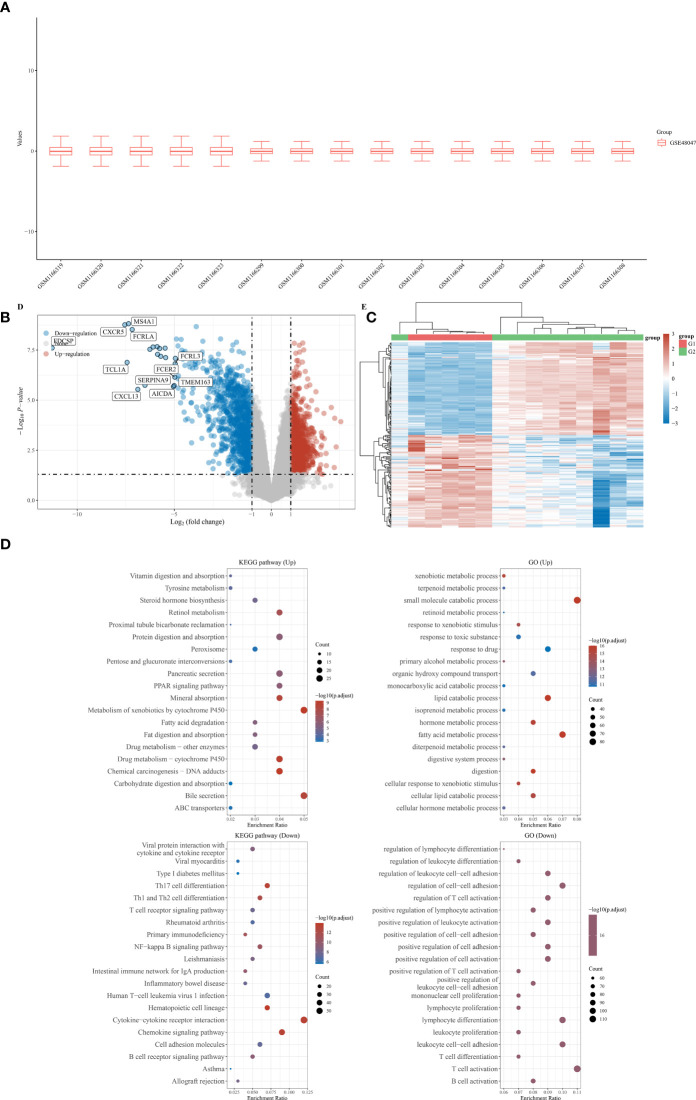
**(A)** The duodenal tissue lymphoma tissues and normal duodenal tissues were shown in the boxplot; **(B)** The differentially expressed analysis between duodenal tissue lymphoma tissues and normal duodenal tissues; **(C)** The heat map was performed to evaluated the differentially expressed between duodenal tissue lymphoma tissues and normal duodenal tissues; **(D)** The GO and KEGG analysis based on the differentially expressed genes.

First, a total of five normal stomach tissues and 10 stomach tissues of gastric malt lymphoma patients were involved in the GSE48047 cohort in the GEO database. The differential expression analysis between gastric MALT lymphoma patients and control groups was performed in R. The results demonstrated that a total of 2329 differential expressed genes were screened in the gastric malt lymphoma patients compared with the normal people’s stomach tissue. 685 of them were up-regulated genes and 1644 of them were down-regulated (**Figures 1A-C**). The GO and KEGG enrichment analysis demonstrated that many enriched pathways, including Vitamin digestion and absorption, steroid hormone biosynthesis, xenobiotic metabolic process, uronic acid metabolic process, urea metabolic process, small molecule catabolic process, fatty acid metabolic process and regulation of leukocyte mediated immunity (**Figure 1D**). In addition, the GSE48047 cohort also contains the expressed data of duodenal tissue lymphoma tissues. We then performed the differential expressed analysis based on the data between duodenal tissue lymphoma tissues and normal duodenal tissues. The results revealed that a total of 684 differential expressed genes were detected, which includes 284 up-regulated genes and 400 down-regulated genes (**Figures 2A-C**). Subsequently, the GO and KEGG enrichment revealed that vitamin digestion and absorption, steroid hormone biosynthesis, small molecule catabolic process, response to toxic substances, regulation of leukocyte cell−cell adhesion, regulation of lymphocyte differentiation, regulation of leukocyte differentiation, regulation of leukocyte cell−cell adhesion, positive regulation of lymphocyte activation, and lymphocyte proliferation (**Figure 2D**).

### Exploration of the genes that are associated with DLBCL and gastrointestinal MALT lymphoma

**Figure 3:**
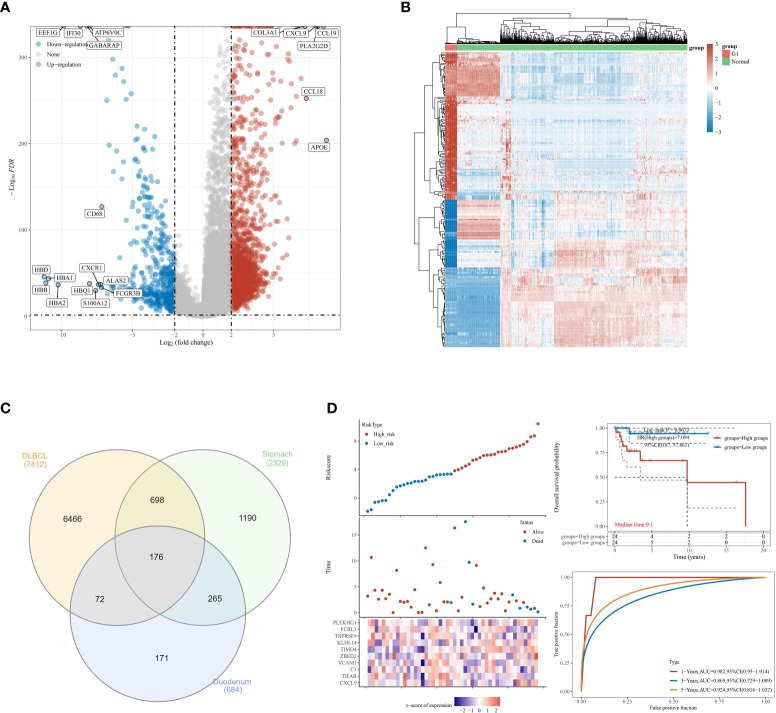
**(A)** The differential expressed analysis based on the DLBCL cohort in TCGA database; **(B)** The heat map reveals the differentially expressed genes based on the DLBCL cohort in TCGA database; **(C)** The venn diagram was applied to explore co differentially expressed genes involved in TCGA and GEO database; **(D)** The construction of the prognostic prediction model in DLBCL cohort.

Most gastrointestinal lymphomas are of B-cell origin, and DLBCL is the most common. Therefore, we then explore the relationship between DLBCL and gastrointestinal MALT lymphoma. By using the TCGA database, we obtain the differential expressed genes in patients with the high and low stage of DLBCL. The results demonstrated that a total of 7412 genes were considered as differential expressed genes, which includes 2695 up-regulated genes and 4717 down-regulated genes (**Figures 3A, B**). Subsequently, in order to explore the genes that are closely associated with DLBCL and gastrointestinal MALT lymphoma. We then performed the Venn Diagram, the results demonstrated that 176 intersection genes were obtained in DLBCL lymphoma cohort and gastrointestinal MALT lymphoma cohort (**Figure 3C**).

### Construction of the prognostic prediction in DLBCL cohort based on the 17 intersection genes

To further explore the genes that are closely related to the prognosis of DLBCL lymphoma patients, we then performed the lasso regression analysis based on the expression of genes and the overall survival rate of DLBCL patients. We finally obtained 10 genes that are closely associated with the prognosis of DLBCL patients, including CXCL9, TIFAB, C3, VCVAM1, ZBE, D2, TIMD4, KLHL14, TNFRSF9, FCRL3, and PLEKHG1. For DLBCL patients, the Risk score = (0.0411) * CXCL9 + (-0.7688) * TIFAB + (0.0489) * C3 + (2.0974) * VCAM1 + (0.5684) * ZBED2 + (1.3951) * TIMD4 + (0.165) * KLHL14 + (-3.1688) * TNFRSF9 + (-0.4584) * FCRL3 + (0.1244) * PLEKHG1. Based on the expressed level of these ten genes, the patients with DLBCL were divided into the low and high-risk groups. The survival analysis demonstrated that the OS of DLBCL patients in the low-risk group is significantly higher than patients in the high-risk group. In addition, the ROC curve reveals that the risk score showed good predictive value in the prognosis of DLBCL patients. The AUC score of ROC for 1-year, 3-year, and 5-year was 0.982, 0.869, and 0.924 respectively. The AUC score is much larger than 0.7, which aims that the prognostic prediction model has good predictive value for DLBCL patients (**Figure 3D**).

### Detection of the mutation frequency of 10 genes that are involved in prognostic prediction model in DLBCL cohort

**Figure 4:**
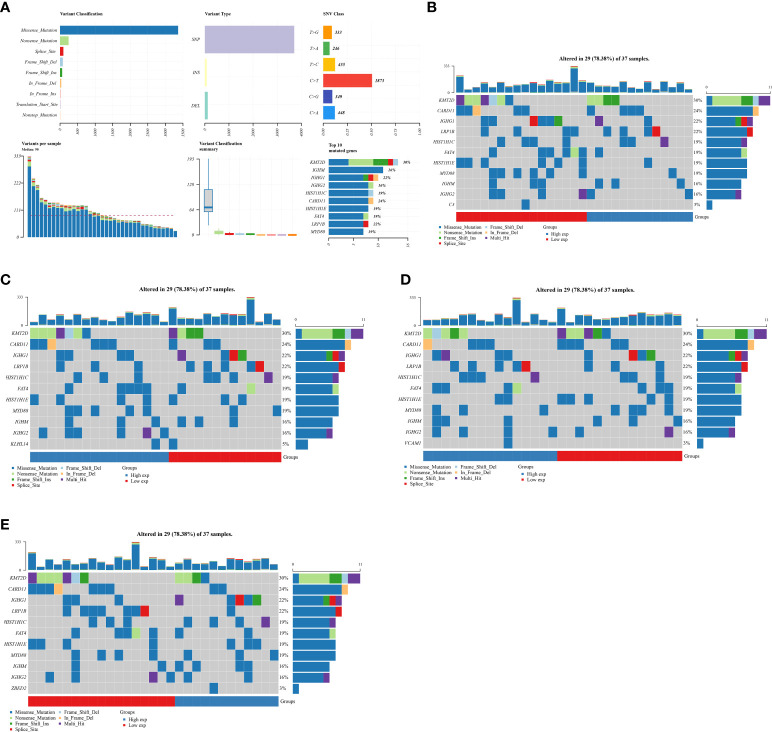
**(A)** The total gene mutation landscape in DLBCL cohort; **(B)** The mutation landscape of C3 in DLBCL cohort; **(C)** The mutation landscape of KLHL14 in DLBCL cohort; **(D)** The mutation landscape of VCAM1 in DLBCL cohort; **(E)** The mutation landscape of ZBED2 in DLBCL cohort.

Subsequently, to evaluate the genes that are closely associated with DLBCL patients, we then evaluated the mutation frequency of 10 genes that are involved in prognostic prediction model in DLBCL cohort (**Figure 4A**). The results revealed that four genes of them showed mutations in the DLBCL cohort, including C3, KLHL14, VCAM1 and ZEBD2. However, in the DLBCL cohort, CXCL9, TIFAB, TIMD4, TNFRSF9, FCRL3 and PLEKHG1 did not show mutation. The mutation rate of C3, VCAM1 and ZEBD2 in somatic cells is three percent. For KLHL14, the mutation rate of somatic cells is five percent (**Figures 4B-E**).

### Differences in immune cell infiltration and immunotherapy related to different expression of C3, KLHL14, VCAM1 and ZEBD2

**Figure 5:**
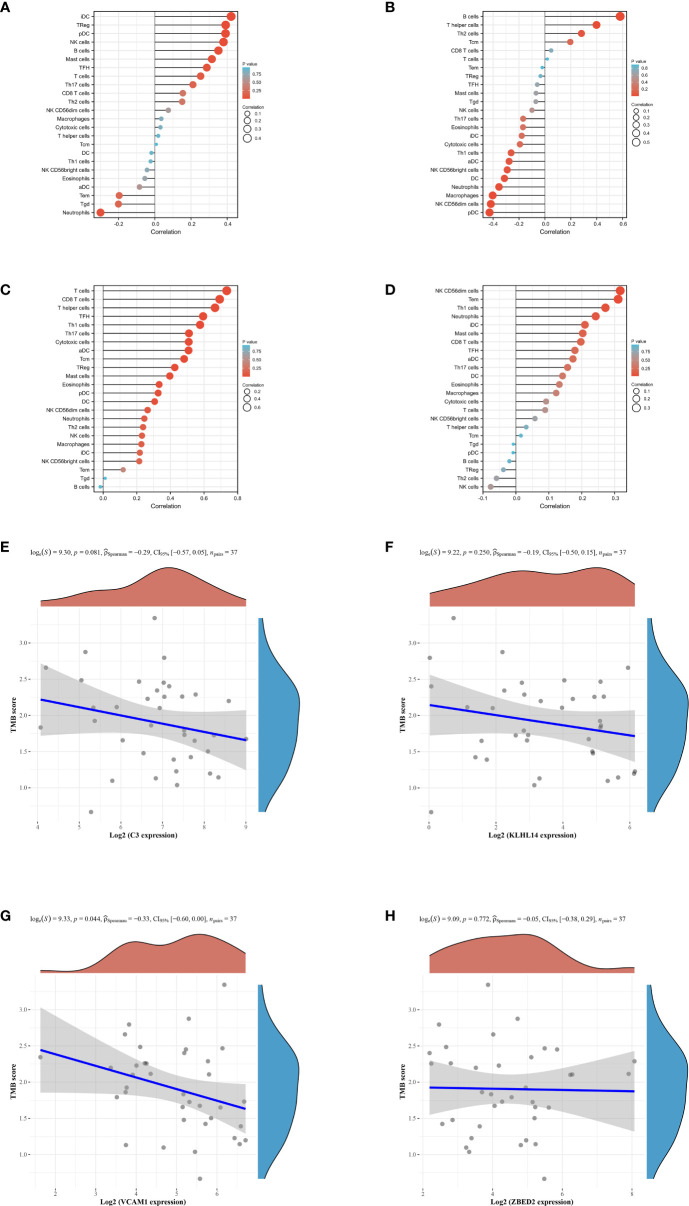
**(A)** The immune cell infiltration of C3 in DLBCL cohort; **(B)** The immune cell infiltration of KLHL14 in DLBCL cohort; **(C)** The immune cell infiltration of VCAM1 in DLBCL cohort; **(D)** The immune cell infiltration of ZBED2 in DLBCL cohort; **(E)** The correlation between the expression level of C3 and TMB score; **(F)** The correlation between the expression level of KLHL14 and TMB score; **(G)** The correlation between the expression level of VCAM1 and TMB score; **(H)** The correlation between the expression level of ZBED2 and TMB score.

Our next step was to examine differences in immune cell expression between the groups. A significant difference was observed between high and low risk groups in the expression of C3, KLHL14, VCAM1 and ZEBD2, which may be the potential targets for immunotherapy. The results demonstrated that the expression level of C3 is positively correlated with the Idc, treg, pdc, NK cells, B cells and mast cells (**Figure 5A**). For KLHL14, the analysis immune cell demonstrated that B cells, T helper cells, The cells and Tcm are positively correlated with the expression level of KLHL14 (**Figure 5B**). In terms of VCAM1, the results revealed that T cells, CD8 T cells, T helper cells, TFH, Th1 cells, Th17 cells, adc and tcm are positively correlated with the expression level of VCAM1 (**Figure 5C**). The expression level of NK cells, tem, th1 cells, idc and mast cells are positively correlated with the expression level of VCAM1 (**Figure 5D**). Tumor mutational burden (TMB) is of interest in immunotherapy, and PDL1 is an important biomarker for predicting response to PD1 antibody therapy. Subsequently, we evaluate the relationship between C3, KLHL14, VCAM1, ZEBD2 and TMB. The results demonstrated that the expression levels of C3, KLHL14, VCAM1 and ZEBD2 are negatively correlated with TMB, which suggested that C3, KLHL14, VCAM1 and ZEBD2 may be important biomarkers for predicting response to PD1 antibody therapy (**Figures 5E-H**).

### Exploration of the role of KLHL14 in immune scores, immune checkpoints and ICB responses

**Figure 6:**
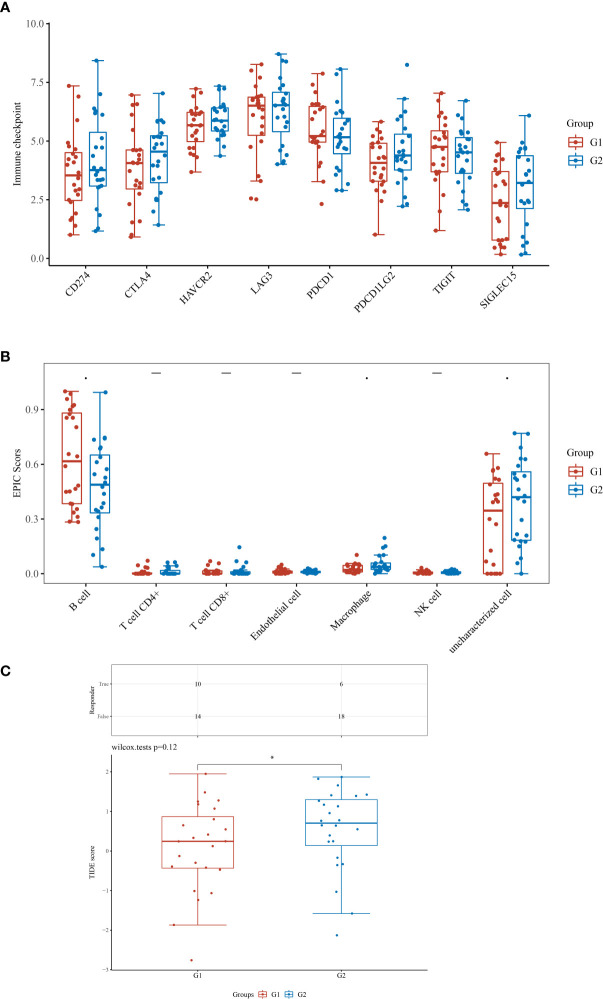
**(A)** The different immune score between low- and high- KLHL14 group; **(B)** The immune check point between low- and high- KLHL14 group; **(C)** The different TIDE score between low- and high- KLHL14 group.

Based on the prognostic prediction model and the tumor mutation analysis, we discovered that KLHL14 may play an important role in DLBCL cohort. Therefore, we then evaluated the role of KLHL14 in the immune scores, immune checkpoints and ICB responses of DLBCL cohort (**Figures 6A-C**). The results of immune checkpoints demonstrated that the expression levels of CD24, CTLA4, PDCD1LG2 and SIGLEC15 are correlated with the expression of KLHL14. For B cells, the KLHL14-high expression group may be associated with more B cells. However, the KLHL14-high may also be associated with less macrophage. In addition, DLBCL patients with higher expression of KLHL14 may be associated with better immunotherapy efficacy.

### Function enrichment analysis of KLHL14 in DLBCL patients

**Figure 7:**
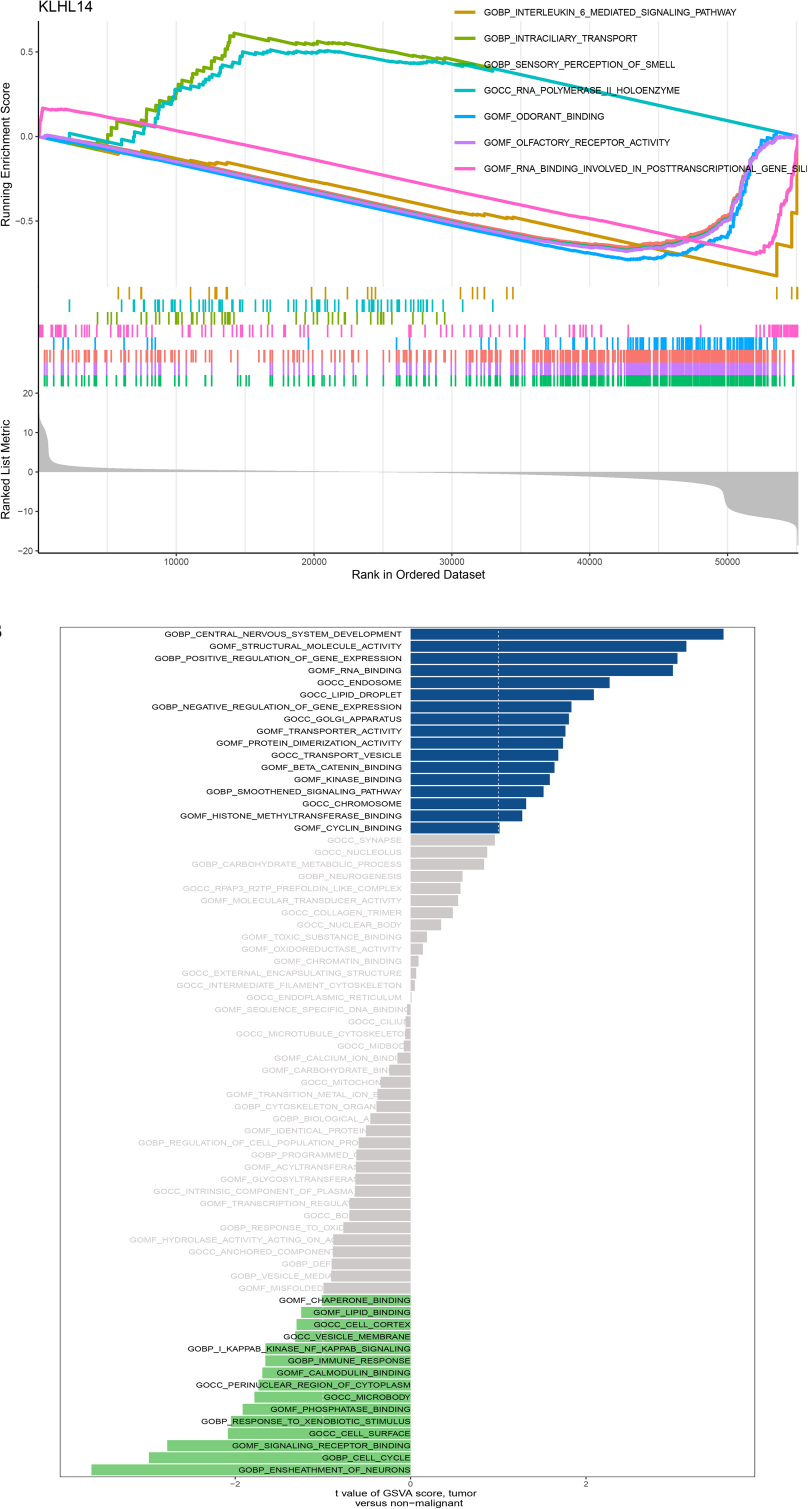
**(A)** The GSEA enrichment of KLHL14 in DLBCL cohort; **(B)** The GSVA enrichment analysis of KLHL14 in DLBCL cohort.

The results of GSEA enrichment analysis demonstrated that some pathways, including sensory perception of smell, olfactory receptor activity, detection of chemical stimulus, odorant binding, RNA binding involved in posttranscriptional gene silencing, intraciliary transport and DNA-dependent ATPase activity were closely associated with KLHL14 (**Figure 7A**). In addition, for GSVA enrichment analysis, the results demonstrated that sheath of neurons, central nervous system development, structural molecule activity, positive regulation of gene expression, RNA binding, cell cycle, signaling receptor binding, transporter activity and immune response were positively correlated with the expression level of KLHL14 (**Figure 7B**).

## Discussion

MALT lymphoma of the stomach and duodenum is a rare tumor that arises from mucosa-associated lymphoid tissue, usually caused by chronic Helicobacter pylori infection (18). Most gastric lymphomas are MALT lymphomas, which make up 38%-48% of all primary gastric lymphomas (19). The majority of gastric MALT lymphomas are located locally and are usually of low grade. MALT lymphoma is most likely to transform into high-grade DLBCL, although this is rarely the case (20). Therefore, in this work, we aim to explore the risk factors for gastrointestinal MALT lymphoma in developing DLBCL. Firstly, by screening the GEO database, we obtain the transcriptome expression data of normal stomach, duodenum tissues and gastric, duodenal MALT lymphoma tissues. The differential expressed analysis provided us with the genes that play an important role in the development of gastric and duodenal MALT lymphoma. Subsequently, in order to further explore the genes that are closely associated with the DLBCL, we then explore the expression data in DLBCL cohort, the Venn Diagram demonstrated that 176 of differential expressed genes are closely associated with the DLBCL and gastric, duodenal MALT lymphoma. To explore the genes with high risk factors in DLBCL patients, we then construct the prognostic prediction model based on the gene expression level of the overall survival rate of DLBCL patients. We finally obtained ten key genes that are closely associated with the prognosis of DLBCL patients, including CXCL9, TIFAB, C3, VCVAM1, ZBE, D2, TIMD4, KLHL14, TNFRSF9, FCRL3 and PLEKHG1. The survival analysis demonstrated that ten genes based prognostic prediction model is closely associated with the overall survival rate of DLBCL patients. In addition, the ROC curve also revealed that ten genes based prognostic prediction model has good prognostic prediction value in DLBCL patients. Approximately 40% of adults develop diffuse large B-cell lymphoma, which is the most common type of non-Hodgkin lymphoma (21). Patients with diverse DLBCLs have variable prognoses and responses to treatment because of their heterogeneous pathologies. It is therefore essential to conduct gene expression profiling to gain a comprehensive understanding of DLBCL (22). There have been some studies identifying key genes in DLBCL, including EZH2, FOXP1, and TP53, but the cause of the disease in the gastrointestinal tract has not been determined (23). Due to its blast cells, pathological characteristics, and pathogenic genes, gastrointestinal DLBCL differs somewhat from DLBCL. Therefore, we focused on the genes that play an important role in the gastrointestinal DLBCL cohort in this work (24).

The GO and KEGG enrichment analysis based on the differential expressed genes demonstrated that some immune-related genes were closely associated with immune, including immune response−activating signal transduction, immune response−activating cell surface, intestinal immune network for IgA production, primary immunodeficiency, lymphocyte proliferation and lymphocyte differentiation. For better outcomes, several phase III trials utilizing rituximab and CHOP (R-CHOP) have been performed over the past several decades (24). With this approach, 50-70% of DLBCL patients are usually cured, thus becoming the standard treatment for the disease. It is important to note, however, that other patients who require replacement therapy have a poor prognosis, and most of them will eventually die. Therefore, it is important to explore better immunotherapy for gastrointestinal DLBCL patients (25).

In addition, in order to explore the genes that are most correlated with gastrointestinal DLBCL patients, we then performed to detect the mutation frequency, immune cell infiltration, and immunotherapy of the ten key genes in the DLBCL cohort. The results revealed that the mutation rate of KLHL14 in somatic cells is five percent. Further, For KLHL14, the analysis of immune cells demonstrated that B cells, T helper cells, The cells, and Tcm are positively correlated with the expression level of KLHL14. Also, the response of immunotherapy in gastrointestinal DLBCL patients is closely associated with KLHL14. The former study demonstrated that KLHL14 is frequently inactivated in mature B-cell malignancies, especially in diffuse large B-cell lymphomas, whose survival is dependent on the signaling of B-cell receptors26. In another study, KLHL14 was found to be a predictive factor of poor prognosis in patients with ovarian cancer and a target for early detection. A significant level of KLHL14 expression was observed in ovarian cancer patients at all stages (26). In this work, KLHL14 was considered as a good marker in gastrointestinal DLBCL patients.

Finally, we explored the potential pathways that are closely associated with KLHL14, the results revealed that KLHL14 is highly correlated with lymphoma. Some pathways, include lymphocyte proliferation and lymphocyte differentiation. In addition, the immune-related pathways, such as immune response, immunoglobulin receptor binding, and immunoglobulin complex circulating were also closely associated with KLHL14. Blocking the PD-1 pathway in DLBCL has led to regulatory approval, and clinicians are eager to assess their utility early in the course of the disease. The development of reliable biomarkers to identify which patient subgroups are likely to benefit from checkpoint blockade is therefore necessary for lymphoma subtypes with limited response. To follow up on these observations, prospective clinical studies using immune checkpoint inhibitors are being conducted. It is important to develop reliable biomarkers in lymphoma subtypes with a limited response to checkpoint blockade to predict which subgroup of patients will benefit from these drugs.

## Data availability statement

The original contributions presented in the study are included in the article/supplementary material. Further inquiries can be directed to the corresponding author.

## Author contributions

XJ and HZ contributed equally to this work. All authors contributed to the article and approved the submitted version.
